# The use of technology to support lifestyle interventions in knee osteoarthritis: A scoping review

**DOI:** 10.1016/j.ocarto.2023.100344

**Published:** 2023-02-09

**Authors:** David F. Hamilton, Shehnaz Akhtar, Benjamin Griffiths, Yeliz Prior, Richard K. Jones

**Affiliations:** a Research Centre for Health, Glasgow Caledonian University, Glasgow, UK; b School of Health and Society, Centre for Human Movement and Rehabilitation, University of Salford, Salford, UK

**Keywords:** Knee, Osteoarthritis, Technology, Lifestyle interventions

## Abstract

**Introduction:**

Technological tools that promote the adoption of physical activity to increase individuals’ functional ability in knee osteoarthritis (OA) are desired to support lifestyle interventions. However, there is little consensus as to the current use of such supportive interventions for knee OA. The aim of this scoping review is therefore to provide an overview on the current use of technology within lifestyle interventions for individuals with knee OA.

**Methods:**

Scoping review as per PRISMA guidance. Structured search of Cochrane Central Register for Controlled Trials, ELSEVIER, IEEExplore, GOOGLE Scholar, MEDLINE, PEDRO, PUBMED, WEB OF SCIENCE from 2010 to 2020 inclusive. Hits were screened by title and abstract and then full text review based on pre-defined criteria. Results were synthesised and pooled by theme for reporting.

**Results:**

2508 papers were identified, and following review, 78 studies included. Papers included interventions for individuals with knee osteoarthritis (n ​= ​31), total or partial knee arthroplasty (n ​= ​20) and developmental work in healthy controls (n ​= ​27). Of the 78 studies, 47 were carried out in laboratory settings and 31 in the field. The identified themes included Movement measurement (n ​= ​24), Tele-rehabilitation (n ​= ​22), Biofeedback (n ​= ​20), Directly applied interventions (n ​= ​3), Virtual or augmented reality (n ​= ​5) and Machine learning (n ​= ​4).

**Conclusions:**

The predominant current use of technology in OA lifestyle interventions is through well-established telecommunication and commercially available activity, joint angle and loading based measurement devices, while integrating new advanced technologies seems a longer-term goal. There is great potential for the engineering and clinical community to use technology to develop systems that offer real-time feedback to patients and clinician as part of rehabilitative interventions to inform treatment.

## 1 Introduction

Osteoarthritis (OA) is the most common joint disease worldwide, affecting an estimated 10% of men and 18% of women over 60 years of age [1]. Specifically, knee OA (KOA) ranks highly among the global causes of disability and is responsible for substantial health and societal costs [2]. It has a multifactorial aetiology but is broadly considered the product of an interplay between systemic and local factors associated with disease onset and mechanical/traumatic facilitators.

There is no cure for OA, only symptom mitigation strategies. End-stage disease can be addressed with surgery and in 2019, 103,617 knee replacements were carried out in England and Wales alone, of which 98% were attributed to OA [3]. However, surgery is not without risk and should only be considered having exhausted non-surgical management options. KOA is also associated with greater prevalence of cardiovascular disease where sufferers are three times as likely to have heart failure or ischemic heart disease compared with matched non–KOA cohorts [4]. Additionally, KOA significantly limits a person's ability to self-manage other conditions, such as diabetes, and hypertension given that KOA related pain is associated with reduced physical activity [5]. As such, encouraging physical activity through lifestyle interventions is particularly important for managing symptoms of the disease and associated comorbidities and increasing function.

Self-management incorporating physical activity, maintaining and reducing body weight, reducing sedentary time and addressing other health risk factors such as diabetes management, has been found to be effective at improving both functional outcomes [6] and symptoms of KOA [7]. However, at the population level, mixed results have been achieved [8]. This is most likely due to a failure of behaviour modification leading to a lack of long-term adoption of these interventions and a return to reduced physical activity levels. The WHO Highlights that access to information on functional exercises, pain management is limited and adherence to such interventions remains a challenge [9].

Various products are sold directly to patients to massage, heat, cool or stabilise the knee joint. There is a need though for tools and/or interventions that promote the adoption of physical activity to increase individuals’ functional abilities and reduce associated pain. Commonly available technology to facilitate this includes smartphones and watches or other Internet-enabled devices to prompt and monitor interventions. However, there is surprisingly little research as to the current use of supportive interventions within KOA treatments. One relatively recent systematic review [10] examined the use of wearable technology from the perspectives of persons with osteoarthritis and found only 7 research papers addressing this subject.

Given the ability for technology to enhance lifestyle interventions there is a clear need for a broader understanding of how technology is currently used within the KOA setting. The use of technology within clinical interventions provides an opportunity to further enhance self-management strategies. In addition to communication devices and apps to guide treatments, this could include more direct biomechanical evaluation and correction using lab-based equipment or wearable devices and direct therapeutic interventions. Technology is rapidly evolving, and development of advanced technologies is increasing at an exponential rate. However, the development of technology within healthcare is often slow to be embraced. Typically, only enthusiastic clinicians collaborating with academia pursue leading technology, which results in slow progress of both technology adoption and health outcomes.

Anecdotally, technologies are being increasingly utilised to facilitate lifestyle interventions within KOA populations but there is ambiguity as to the types of available technology, applications of this, and the potential of developing technologies. Therefore, this scoping review aims to address this knowledge gap, map the literature, and provide an overview as to the current use of technology within lifestyle interventions for individuals with knee OA by identifying the key concepts and sources of evidence that inform practice in the field.

## 2 Methods

This was a scoping project aiming to survey and summarise the existing literature as to the use of technology in KOA lifestyle interventions. We followed a systematic approach, which was informed by the extended PRISMA guidance for conducting systematic reviews, and based on the framework for conducting scoping reviews set out by Arksey and O'Malley [11] and advanced by Daudt, Van Mossel, and Scott [12]. The primary research question was ‘what is the current use of technology supporting lifestyle interventions for the management of KOA?’ which was developed as per the population, concept and context (PCC) model which is appropriate for scoping review questions [13].

The search strategy was developed by a research team compromising methodologists and a specialist librarian. A wide literature search was performed including keywords and MESH terms. The following eight databases were searched: Cochrane Central Register for Controlled Trials, ELSEVIER, IEEExplore, GOOGLE Scholar, MEDLINE, PEDRO, PUBMED, WEB OF SCIENCE.

The three-step search process advocated by the Joanne Briggs Institute was followed. Firstly, an initial search was carried out within two databases MEDLINE and PsycINFO to allow keywords checking and the Medical Subject Headings (MeSH) that appeared in the results from this initial search were applied. Secondly, supplementary synonyms for keywords were added to the search terms. Thirdly, references from included articles were searched followed by a final search of Google Scholar, with a limitation as to the cut of date ensuring consistency with the previous searches.

We incorporated searches around “knee joint” AND “osteoarthritis,” AND “technology “AND “lifestyle intervention”: (Search 1) (“Lifestyle-intervention” [All Fields] (Lifestyle OR behaviour OR conduct OR habits OR style of living OR style of habits [All Fields]); (Search 2) AND (Intervention OR interference OR mediation OR arbitration OR intercession OR interposition OR interruption [All Fields]); (Search 3) AND (technology [All Fields]); (Search 4) AND (“Knee osteoarthritis” OR “knee joint” [All Fields]) AND (Osteoarthritis [All Fields]). Additional relevant articles were sought through manual searching of reference lists of identified literature. The retrieved articles were stored in a Mendeley web-library and reviewed within an Endnote library.

Articles were selected by a process of title and abstract screening leading to full text review by two independent reviewers with inclusion by agreement. A third independent reviewer was available as arbitrator to resolve any conflicts. The articles were compared against the inclusion criteria and screened for significance. The relevant studies were included if they were published between 2010 and 2020, a timeframe chosen to reflect the current use of technology within KOA lifestyle interventions and reflected the use of technology to support a lifestyle intervention for individuals with KOA. All types of quantitative study designs were included and extended conference publication accepted, so long as these reported full text papers. Data from each article was recorded into a table including author/publication details, study aims, population, intervention, key findings relevant to the review question and the intervention location (laboratory vs free-living). Following review, the articles were categorised into themes by the study team, based on the technology focus of a collection of similar articles. Methodological quality of included papers is not assessed but a narrative overview of content provided.

## 3 Results

### 3.1 Study selection

**Fig. 1:**
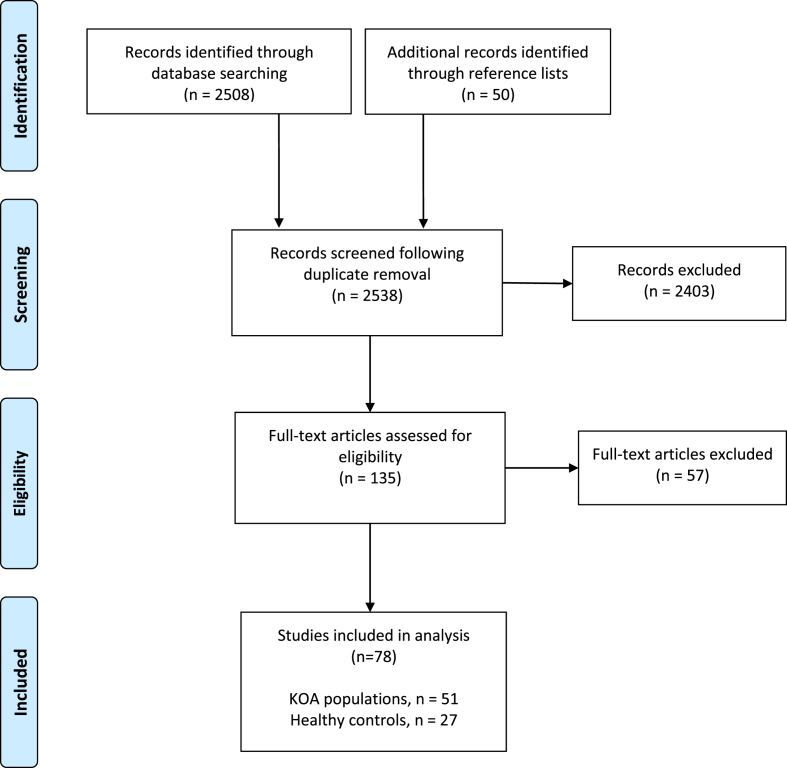
Scoping review PRISMA flowchart.

2508 papers were identified across the eight databases and an additional 50 through the reference lists of eventually included articles. Following removal of duplicates, 2538 articles were screened by title and abstract, of which 135 papers were eligible for full text review. Papers were excluded due to not describing the application of technology to a KOA lifestyle intervention. Of the 135 full text papers reviewed, 57 were deemed not to be relevant to this review as the technology described in the work could not be applied in the context of a KOA intervention, resulting in 78 articles in the final selection (see Fig. 1).

Of the included studies, 51 articles reported interventions in KOA populations and 27 articles focused on the development or assessment of a technology that was explicitly designed for a KOA intervention. The participants in these developmental studies were healthy controls or contributors who had no symptoms of KOA. These studies were included on the basis that this constitutes emerging technology that are specifically intended to be used within KOA interventions, which brought it within the scope of our predefined review criteria.

### 3.2 Study characteristics

The papers discussed interventions for individuals with KOA (n ​= ​31), applications for total or partial knee arthroplasty (n ​= ​20) and the remainder development of technology in healthy controls (n ​= ​27). Of the 78 studies, 47 were carried out in laboratory settings and 31 in the field. The processes of identification of KOA in the included studies varied from self-reported symptoms to radiographic evidence of KOA, while some papers did not disclose or discuss inclusion criteria. Each paper was categorised into themes based on the main technological intervention described within the text. Many interventions used multi-modal technologies, we categorised these papers based on their primary outcome or main discussion points. We report various statistical values (e.g. percentages, correlation co-efficients, significance tests) in our narrative summary of the included papers where relevant and when this information was reported in the included papers.

### 3.3 Study themes

We identified six technology themes including; Movement measurement (n ​= ​24), Tele-rehabilitation (n ​= ​22), Biofeedback (n ​= ​20), Directly applied interventions (n ​= ​3), Virtual or augmented reality (n ​= ​5) and Machine learning (n ​= ​4). Individual study details including population, device themes and settings are provided in Table 1.

**Table 1:** Included studies: populations and interventions.

Publication Details		Intervention	Population		
Author	Date	Primary technology	Type	Size	Location
Atallah et al. [14]	2011	IMU	TKA	8	Lab
He et al. [15]	2019	IMU	KOA	6	Lab
Giggins et al. [16]	2014	IMU	KOA	18	Lab
Jeong et al. [17]	2019	IMU	KOA	52	In-field
Lee et al. [18]	2015	IMU	KOA	1168	In-field
Li et al. [19]	2018	IMU	KOA	61	In-field
Ashapkina et al. [20]	2018	IMU	Healthy	n/a	Lab
Bevilacqua et al. [21]	2018	IMU	Healthy	n/a	Lab
Charlton et al. [22]	2018	IMU	Healthy	8	Lab
Xia et al. [23]	2017	IMU	Healthy	14	Lab
Chen et al. [24]	2017	IMU	Healthy	10	Lab
Ishake et al. [25]	2017	IMU	Healthy	2	Lab
Zexia He et al. [26]	2017	IMU	Healthy	1	Lab
Ficklsherer et al. [27]	2016	Force Transducer	TKA	30	Lab
Fung et al. [28]	2012	Force Transducer	TKA	50	Lab
Mcclelland et al. [29]	2012	Force Transducer	TKA	1	Lab
Zeni et al. [30]	2013	Force Transducer	TKA	11	In-field
Christiansen et al. [31]	2015	Force Transducer	TKA	26	Lab
van Den Noort et al. [32]	2011	Force Transducer	KOA	20	Lab
Ferrigno et al. [33]	2016	Force Transducer	Healthy	32	Lab
Kang et al. [34]	2013	Goniometry	Healthy	7	Lab
Kang et al. [35]	2019	Goniometry	KOA	10	Lab
Rickowski et al. [36]	2010	Goniometry	Healthy	15	Lab
Bergmann et al. [37]	2013	Goniometry	Healthy	10	Lab
Dobson et al. [38]	2014	Tele-rehabilitation	KOA	n/a	In-field
Moffet et al. [39]	2015	Tele-rehabilitation	TKA	205	In-field
Russel et al. [40]	2011	Tele-rehabilitation	TKA	65	In-field
Tousignant et al. [41]	2011	Tele-rehabilitation	TKA	48	In-field
Bennell et al. [42]	2017	Tele-rehabilitation	KOA	148	In-field
Bini et al. [43]	2016	Tele-rehabilitation	TKA	29	In-field
Rini et al. [44]	2015	Tele-rehabilitation	KOA	113	In-field
Chughtai et al. [45]	2019	Tele-rehabilitation	TKA	157	In-field
Eichler et al. [46]	2019	Tele-rehabilitation	TKA	111	In-field
Piqueras et al. [47]	2013	Tele-rehabilitation	TKA	142	In-field
Smittenaar et al. [48]	2017	Tele-rehabilitation	KOA	41	In-field
Tipprom et al. [49]	2018	Tele-rehabilitation	KOA	6	Lab
Correia et al. [50]	2018	Tele-rehabilitation	TKA	59	In-field
Bennell et al. [51]	2017	Tele-rehabilitation	KOA	168	In-field
Allen et al. [52]	2010	Tele-rehabilitation	KOA	515	In-field
O'Brien et al. [53]	2018	Tele-rehabilitation	KOA	120	In-field
Nelligan et al. [54]	2019	Tele-rehabilitation	KOA	128	In-field
Klement et al. [55]	2019	Tele-rehabilitation	TKA	296	In-field
Dar et al. [56]	2014	Tele-rehabilitation	KOA	14	In-field
Bossen et al. [57]	2013	Tele-rehabilitation	KOA	199	In-field
Beukenhorst et al. [58]	2020	Tele-rehabilitation	KOA	26	In-field
Skrepnik et al. [59]	2017	Tele-rehabilitation	KOA	111	In-field
Hengsomboon et al. [60]	2019	Sensory feedback	KOA	52	Lab
Routson et al. [61]	2016	Sensory feedback	KOA	10	Lab
Dowling et al. [62]	2010	Sensory feedback	Healthy	9	Lab
Wheeler et al. [63]	2011	Sensory feedback	Healthy	16	Lab
Shull et al. [64]	2011	Sensory feedback	Healthy	9	Lab
Chen et al. [65]	2015	Sensory feedback	Healthy	10	Lab
Shull et al. [66]	2010	Sensory feedback	Healthy	10	Lab
Lurie et al. [67]	2011	Sensory feedback	Healthy	9	Lab
Hunt et al. [68]	2011	Motion capture	KOA	15	Lab
Hunt et al. [69]	2014	Motion capture	KOA	20	Lab
Richards et al. [70]	2018	Motion capture	KOA	16	Lab
Richards et al. [71]	2018	Motion capture	KOA	40	Lab
van Den Noort et al. [72]	2015	Motion capture	Healthy	17	Lab
Jackson et al. [73]	2018	Motion capture	Healthy	11	Lab
Barrios et al. [74]	2010	Motion capture	Healthy	8	Lab
Jun et al. [75]	2013	Motion capture	Healthy	5	Lab
Akkaya et al. [76]	2012	EMG	PKA	45	Lab
Yilmaz et al. [77]	2010	EMG	KOA	40	In-field
Wang et al. [78]	2015	EMG	TKA	66	In-field
Pizzolato et al. [79]	2017	EMG	Healthy	5	Lab
Bruce-Brand et al. [80]	2012	NMES	KOA	41	In-field
Palmieri-Smith et al. [81]	2010	NMES	KOA	40	In-field
Walls et al. [82]	2010	NMES	TKA	14	In-field
Argent et al. [83]	2019	Virtual reality	TKA	15	In-field
Su et al. [84]	2015	Virtual reality	TKA	27	Lab
Gonzalez-Franco et al. [85]	2014	Virtual reality	Healthy	16	Lab
Qui et al. [86]	2017	Virtual reality	Healthy	n/a	Lab
Karatsidis et al. [87]	2018	Virtual reality	Healthy	11	Lab
Favre et al. [88]	2012	Machine learning	KOA	28	Lab
Hunt et al. [89]	2011	Machine learning	KOA	47	Lab
Chen et al. [90]	2016	Machine learning	Healthy	10	Lab
Taylor et al. [91]	2010	Machine learning	Healthy	6	Lab

KOA: Knee Osteoarthritis, TKA: Total Knee Arthroplasty, PKA: Partial Knee Arthroplasty, IMU: Inertial Measurement Unit, NMES: Neuromuscular Electrical Stimulation, EMG: Electromyography.

#### 3.3.1 Theme 1 - movement measurement technologies

Studies that employed movement measurement technologies as a lifestyle intervention were the largest category in the review (n ​= ​24), however many of these (n ​= ​10) were at the developmental stage utilising healthy control participants. The majority of this work investigated the use of inertial measurement units (IMUs) (n ​= ​13) and force transducers (n ​= ​7). Three studies explored the use of electronic goniometers and a single paper evaluated using conductive textiles as a goniometer. The general focus was on assessing activity quality within rehabilitation/exercise programs and evaluating walking kinetics to estimate knee adduction moments (KAM).

##### 3.3.1.1 Inertial measurement units

Six studies used IMUs in participants with KOA. Three assessed activities in-situ evaluating walking [14,15] and rehabilitation exercises [16], while the other three used activity monitors to correlate lifestyle variables with health outcomes [[17], [18], [19]]. The depiction of the IMU devices used ranged from detailed technical descriptions (including transducer combinations, dynamic range, and sampling rate) to more simplistic reports of standard outputs using commercial activity monitors. The three papers that assessed activities in-situ validated IMU-data prediction models against gold standard measures. Atallah et al. [14] used an ear-mounted accelerometer and discrete wavelet analysis to classify stages of rehabilitation following TKA and accurately determined TKAs from controls. He et al. [15] used a shoe mounted IMU to estimate toe-in angle and its effect on KAM, an indicator of medial KOA, and Giggins et al. [16] used three lower-limb mounted IMUs to classify rehabilitation exercises and their success/failure, with 83% accuracy reported. Jeong et al. [17] found that pain, symptoms (KOA), function and muscle strength all correlated higher with daily steps, while Lee et al. [18] reported individuals with KOA spent 2/3 of daily time in sedentary behaviour and had slower gait speed and a lower chair stand rate. Li et al. [19] implemented IMU based data collection within remote counselling sessions focusing on education and feedback on lifestyle variables. This intervention significantly improved outcome parameters (daily steps, activity of daily living and quality of life) compared to baseline.

Seven additional studies used IMUs with healthy controls, with a focus on the technology recognising activities and activity assessment. Two papers describe their methods for activity classification; Ashapkina et al. [20] focused on the application of a dynamic time warping algorithm to classify rehabilitation activities from multiple IMU data, while Bevilacqua et al. [21] proposed a 2-phase approach to classification involving signal segmentation and segment classification to improve activity recognition accuracy. Four papers validated IMU based measurements against a gold standard. Two of these [22,23] validated walking foot progression angles using a shoe mounted IMU and found high agreement based on ICC models (ICC 0.95) [22] and lower average error 1.7± 1deg [23]. Chen et al. [24] and Ishak et al. [25] validated an IMU system's ability to recognise an exercise and determine if the exercise was completed correctly, both studies reported high levels of accuracy in classification and in execution parameters. Finally, Zexia et al. [26] described a gait retraining system that used KAM calculated by pressure sensors controlled through altering FPA, calculated using an IMU. Participants were able to decrease their KAM following FPA guided feedback.

##### 3.3.1.2 Force transducers

Force transducers were used in five studies as part of weight-bearing retraining in patients following Total Knee Arthroplasty (TKA). The Nintendo Wii Balance Board (Nintendo, Redmond, WA), a commercial device designed for use with a games console, was used alongside customised software, off-the-shelf games, or an additional force sensing system. Ficklscherer et al. [27] and Fung et al. [28] used 2-arm designs to examine whether the system was appropriate for use within a rehabilitation program following TKA; both found no significant differences in outcome measures or adverse effects compared to traditional care controls. McClelland et al. [29] presented a case report describing the functional and biomechanical changes in one individual after TKA following a movement-retraining program. Knee motion returned to normal levels, gait improved and more symmetrical knee excursion was reported. These results are supported by Zeni et al. [30], who used movement symmetry biofeedback retraining and reported that individuals had greater knee extension during mid stance and more symmetrical knee movements at 6-months post-TKA following the intervention. Similarly, Christiansen et al. [31] found functional improvements (but not knee extensor moments) in a five-time sit-to-stand-test at 6- and 26-weeks post TKA (p ​= ​0.02), a tendency for improved walking speed (p ​< ​0.07) and increased knee extensor moment during walking at 26-weeks (p ​< ​0.01).

van den Noort et al. [32] explored the influence of an instrumented force shoe on gait patterns in KOA patients. Patients wearing the device showed a decrease in walking velocity and cadence (8%), unchanged stride length, an increase in stance time (13%), stride time (11%) and step width (14%). The gait of individuals with KOA was altered by the increase in shoe height, mass, and a change in sole stiffness. These changes were however in line with normal gait variation and may not be clinically relevant. A further single paper explored the use of force transducers in healthy participants. Ferrigno et al. [33] conducted a proof-of-concept study utilising auditory feedback from pressure-detecting shoe insoles to shift plantar pressure medially to reduce KAM. Participants significantly reduced their peak KAM (p ​< ​0.01) using the pressure insoles.

##### 3.3.1.3 Electro-goniometry

Two papers explored the use of electronic goniometers using an instrumented elliptical machine, a low impact exercise machine for gait rehabilitation. Kang et al. [34] suggested that the system was a suitable way to monitor external KAM reporting significant differences between those with KOA and healthy controls. Further, Kang et al. [35] proposed that knee kinematic variables, which influence knee abduction moment (KAM), were closely associated with ankle kinematics and that ankle retraining using this equipment could also aid rehabilitation. A further paper tested electo-goniometry systems designed for KOA interventions on healthy participants. Riskowski et al. [36] explored the use of a feedback-based gait monitoring knee brace which measured knee joint angles during walking. The system produced significant changes in knee joint angle prior to and at initial contact and peak knee extensor, flexor and adductor moments which led to reduced rates of loading.

A single study explored the use of conductive textiles with healthy participants. This clothing integrated technology creates more comfortable and less intrusive measurement devices. Bergmann et al. [37] presented a sensor made from graphitised carbon black nano-powder and polyurethane that exhibited high electrical conductivity, enabling it to assess knee motions though stretch-resistance. During knee bend exercises they reported the system to be accurate with mean absolute errors of 3° (R2 0.99) compared to a gold standard.

#### 3.3.2 Theme 2 - tele-rehabilitation technologies

Papers that investigated the use of tele-rehabilitation technologies as an intervention were the largest category in which work has been carried out with KOA patient groups (n ​= ​22/51). The largest number of these papers investigated the use of video conferencing systems (n ​= ​7), while others explored multi-model tele-rehabilitation systems (n ​= ​6), messaging services (n ​= ​4), telephone services (n ​= ​3) and symptom tracking systems (n ​= ​2). Most papers used a two-arm study design, comparing the intervention to usual treatment controls (n ​= ​14), while the rest used a single-arm design, comparing post-intervention data to baseline measurements.

The seven studies utilising video conferencing systems provided a remote method of patient-to-clinician contact for conducting physical therapy sessions, pain coping training or wider clinical consultations. All studies compared outcome measures with traditional care controls, however differing results are reported. Three studies [[38], [39], [40]] found these systems to be as effective as traditional in person physical therapy in individuals following knee arthroplasty. However, Tousignant et al. [41] found larger improvements following traditional care (as opposed to a remote digital rehabilitation) two months post-discharge. Three further studies [[42], [43], [44]] found superior pain and functional outcomes in KOA patients following online delivery of pain coping skills training and noted these improvements were maintained at 9-month review.

The multi-model tele-rehabilitation systems typically comprised a combination of physical sensors, motion capture systems, video conferencing systems and delivery platforms. Chughtai et al. [45], Eichler et al. [46], Piqueras et al. [47], Smittenaar et al. [48] and Tipprom et al. [49] all evaluated the use of bespoke systems using instructional avatars, aspects of 3D motion capture using commercial gaming cameras (x-box connect), IMUs and video conferencing for the purpose of providing rehabilitation to individuals following knee arthroplasty. The system compensated for a patient's movement patterns with a predetermined target movement and sent them real-time visual feedback to correct the movement performed. The evaluations conducted were of adherence to the system, time spent performing exercises, system usability and clinical outcome scores. Patients received on average one more follow-up visit using the virtual systems and saw similar improvements in clinical outcome scores compared to traditional care. The systems facilitated increased adherence to physical therapy programs and recorded a large amount of time spent completing exercises. Correia et al. [50] however found superior range of motion-based outcomes compared to traditional physical therapy when evaluating a similar system using IMUs, a real-time mobile biofeedback app and a web-based telecommunication platform.

Telephone services, much like video conferencing systems, provided a remote method of patient-to-clinician contact as a substitute for in-person consultations. Bennell et al. [51] investigated the use of a home exercise program with telephone consultations and found no differences in function (WOMAC) or numerical rating pain scale score compared to normal in-person physical therapy. Similarly, Allen et al. [52] found lower visual analogue pain scale scores compared to a traditional care group and a health education group at 12-months. O'Brien et al. [53] evaluated a telephone-based weight management program in KOA and found the system to be less cost effective than traditional methods. Messaging services focused on encouraging patient adherence to physical rehabilitation programs using either SMS, MMS or emails. Nelligan et al. [54] described the behaviour change theory behind an SMS intervention, while Klement et al. [55] investigating the suitability of an email-based intervention compared to outpatient physical therapy finding 65% of patients were suitable for online physiotherapy with daily emails. Dar et al. [56] found MMS services resulted in non-significant improvements in physical function (WOMAC) compared to a control group that received no encouragement. However, Bossen et al. [57] found automated emails and SMS messages resulted in significant improvements in physical function and self-perceived effect.

Finally, symptom trackers have been described which allow patients to monitor their KOA symptoms using a smart device. Two studies [58,59] found that patients adhered to interventions using the devices and that the monitoring was helpful in understanding and managing their condition.

#### 3.3.3 Theme 3 - biofeedback technologies

Twenty studies investigated the use of biofeedback systems, eight directly applied within KOA lifestyle interventions and 12 developmental studies with healthy controls. The focus of these systems was retraining gait patterns to reduce KAM, and thus impact KOA symptoms. Eight studies utilised sensory feedback, eight motion capture (MOCAP), and four electromyography (EMG).

##### 3.3.3.1 Sensory feedback

Two papers investigated sensory feedback systems as part of KOA interventions. Hengsomboon [60] explored how external sound can influence postural control in elderly individuals with KOA, finding no significant relationship. Routson et al. [61] investigated the use of haptic feedback from a vibro-tactile cane, with results showing the ‘smart’ cane helped users achieve the recommended 15% body weight loading compared to naïve cane use and verbal instructions alone.

In developmental work, feedback systems (n ​= ​6), were used for gait retraining. All papers implemented a form of haptic (vibration) feedback to alter gait parameters. Dowling et al. [62] used haptic feedback to encourage a subtle weight-bearing shift towards the medial side of the foot, which resulted in significant reductions of 14.2% in peak KAM relative to controls. Similarly, Wheeler et al. [63] reported a haptic-based gait retraining system reduced peak KAM by 20.7%. Shull et al. [64] explored the use of personalised data-driven feedback for reducing KAM using three haptic motors. Reductions varied between 29% and 48%. Chen et al. [65] explored the use of a haptic ankle bracelet for retraining both FPA and stance width, reporting that nine out of ten participants were able to retrain their gait for both parameters to within 2° and 39 ​mm respectively. Shull et al. [66] presented a gait retraining system that could alter knee joint loading on healthy individuals at risk of developing early stage KOA. While Lurie et al. [67] explored the use of feedback modality and frequency, highlighting that patients have poor perception of multiple haptic feedback cues, prefer to focus on one motion at a time and require several steps to modify gait.

##### 3.3.3.2 Motion capture

Four papers used MOCAP systems to provide feedback during gait retraining. These systems consisted of multiple cameras positioned with in a laboratory setting with retroreflective markers mounted on the patient, providing a 3D representation of the patient for kinematic analysis. All four papers applied this intervention to individuals with KOA with a single-arm design comparing outcome measures pre- and post-intervention. The aim was to alter foot progression angle during walking to reduce KAM, which has links with progressive medial KOA. Hunt and Takacs [68] observed frontal plane lower limb biomechanics following gait retraining and reported significant reductions in KAM coincided with ipsilateral hip, knee and/or lower spine discomfort. Hunt et al. [69] later explored the use of three different visual feedback methods, a mirror, raw video and real-time feedback and asked participants to increase their toe-out angle during the stance phase of walking. Toe-out performance error was significantly less when using real-time biofeedback (p ​= ​0.03), however the clinical relevance of this difference (two degrees toe-out gait performance error) was questionable may not necessitate the economic cost of real-time biofeedback. Similarly, Richards et al. [70,71] found patients could achieve a targeted FPA angle during real-time biofeedback and that changes were maintained through 6-weeks of toe-in gait retaining. However, the patients were unable to alter their KAM, even with real-time biofeedback, without specific instructions on gait modification techniques.

Four further papers explored the use of MOCAP systems in healthy participants, all of which assessed movement during walking or rehabilitation exercises. Three of these studies [[72], [73], [74]] used laboratory MOCAP systems in an attempt to reduce KAM by providing real-time visual feedback during walking. All saw decreased KAM and peak KAM ranging from 7% to 50% reductions. van den Noort et al. [72] found that the kinematic changes that reduced KAM were increased toe-in, increased step width and decreased hip adduction. However, Jackson et al. [73] found that the altered gait patterns were participant-specific; although noted toe-in to be one of the most used strategies. Barrios et al. [74] reported that dynamic knee alignment changes are maintained at one month after training. Separately, Jun et al. [75] investigated the use of a Kinect camera system (Microsoft) to track movements during a squatting exercise reporting correct classification accuracy of 95.6%.

##### 3.3.3.3 Electromyography

Three papers used EMG to provide feedback on muscle activity during rehabilitation exercises and compared functional and strength outcome measures with traditional care controls. Akkaya et al. [76] reported that the addition of EMG biofeedback during rehabilitation following partial knee arthroplasty increased the rate of recovery with significant improvements in Lysholm score and maximum muscle contraction two-weeks post-surgery. However, Yilmaz et al. [77] found no significant difference in patient reported WOMAC scores or muscle strength by including EMG biofeedback within a strengthening exercise program for patients with KOA. Wang et al. [78] investigated the effectiveness of using EMG biofeedback as a relaxation intervention during continuous passive motion therapy following TKA. Compared to the control group, the intervention group reported significantly less pain (p ​= ​0.001).

Separately Pizzolato et al. [79] report an EMG model of the lower limb to estimate tibiofemoral joint loads and to provide feedback to the participant during walking. With this intervention, five healthy participants were able to adapt their gait to reduce medial tibiofemoral contact forces.

#### 3.3.4 Theme 4 - directly applied intervention technologies

Three papers used neuromuscular electrical stimulation (NMES) during rehabilitation. All stimulated the quadriceps femoris muscle group and compared NMES to traditional-care controls. Outcome measures included walking tests, stair climb tests, chair rise tests, WOMAC scores, muscle strength and muscle cross sectional area. Two studies [80,81] explored the use of NMES for KOA rehabilitation but reported contradicting results. Bruce-Brand et al. [80] found significant improvements in functional capacity in both a resistance-training group and NMES group compared to a control group, that was maintained over 14-weeks. Quadriceps femoris cross sectional area was seen to increase significantly. Conversely, Palmieri-Smith et al. (2010) found no change in muscle strength and activation after a 4-week intervention delivered to women with KOA. Walls et al. [82] explored the use of NMES as pre-surgery rehabilitation for individuals undergoing TKA and reported greater preoperative quadriceps femoris strength, which was associated with increases in walk, stair-climb and chair-rise time (p ​> ​0.05).

#### 3.3.5 Theme 5 - virtual & augmented reality technologies

Two papers explored the use of virtual reality systems in KOA, both assessing the usability and user perceptions of the systems. Argent et al. [83] assessed a prototype biofeedback system that combined IMU's and a tablet computer to display a patient's real-time movements via an avatar. High system usability scores and adherence rates suggest it may offer additional support within a rehabilitation program. Similarly, Su [84] explored how a user's motivation and perception of system usability affected rehabilitation performance in a 3D game-based environment. There was high correlation between performance and motivation evaluation scores (r ​= ​0.87, p ​< ​0.001) and the experimental group showed greater improvements in knee bend angle.

A further two studies used gamification to encourage adherence to rehabilitation exercise programs in healthy controls. Both Gonzalez-Franco et al. [85] and Qiu et al. [86] presented systems that utilise game-based feedback to patients completing rehabilitation exercises, displaying a virtual avatar of the user, controlled through movement. Finally, Karatsidis et al. [87] validated the accuracy of an augmented reality headset which provided visual feedback during walking in a healthy population. The system tracked FPA with an accuracy of 2.4deg, suggesting a potential role in gait retraining.

#### 3.3.6 Theme 6 - machine learning technologies

This theme reports more developmental work around technologies that are less directly applicable in that they are primarily analysis, however the technology described is specifically related to prediction models in KOA and falls within the scope of this review. Two papers explored using machine-learning models to predict KAM in KOA patients. Favre et al. [88] report an artificial neural network with 11 input variables, including ground reaction forces and anthropometric measurements to predict KAM. Similarly, Hunt and Bennell [89] used four input variables within a multiple linear regression algorithm to predict KAM. The aim is to identify patients who are more likely to experience high KAM, which could ultimately assist clinicians in deciding treatment options, whilst repeated assessment could also provide a method for monitoring disease progression.

Two further papers developed machine-learning algorithms in healthy participants. Chen et al. [90] proposed a threshold-based posture classification and online segmentation (multi-layer support vector model) of rehabilitation exercises. Initial posture classification accuracy was reported at 97.9% and segmentation accuracy was 92.7%. Meanwhile, Taylor et al. [91] described a method for assessing exercise quality by building an ‘incorrect exercise’ classifier. Ultimately, this technology could help automatically classify rehabilitation exercise for use within telepresence systems.

## 4 Discussion

As remote and wearable sensor technology develops, there is increasing potential for application of these as lifestyle interventions with which to manage osteoarthritis of the knee. The aim of this scoping review was to survey the contemporary use of such technology. Following a rigorous search, we identified papers reflecting differing technologies that we classified into six themes covering a range of applications from improving the delivery of existing services (such as remote physical therapy), to implementing behavioural change through biofeedback, to physical stimulation of muscle. Seventy-eight papers were included within the review, 51 of which evaluated the use of a technology in people with KOA, and a further 27 describing the development of technologies for clinical KOA populations using healthy controls. In total though, only 40% (n ​= ​31) of studies evaluated the technological interventions during their intended use, the remainder describing methodological development or validation, highlighting the embryonic stage that the technology driven lifestyle intervention field is currently at.

A focus of the current use of technology ‘in the field’ with KOA patients was tele-rehabilitation, where comparatively simple devices, such as telephones, text messaging and video conferencing systems substitute or augment in-person consultations, aiming to save time and improve the cost-effectiveness of service delivery. In small scale studies these are suggested to be effective at increasing adherence to physical therapy programs, improving the outcomes of these programs and increasing the overall number of consultations delivered to patients. More advanced tele-rehabilitation technology combines multiple systems such as IMUs, MOCAP and videoconferencing for monitoring, evaluating and consulting with patients remotely. These complex systems aim to provide an objective method of evaluating patient performance and the quality of the execution of physical therapy exercises, which would otherwise be unavailable to the clinician. The use of these technologies was investigated in six studies, reporting at least equivalent outcomes to traditional in-person delivery. These systems perhaps provide the opportunity for a greater understanding of the patient's physical status through the delivery of remotely collected objective data to both patient and clinician. This ability to collect data for movement or muscle performance as part of tele-rehabilitation remains in its infancy, however, the technology enabled collection of real-time performance data during tele-rehab offers the potential to deliver ‘true’ virtual rehabilitation facilitated by a physical therapist, modifying interventions in real time based on the patient's physical responses, as opposed to the more typically employed video consultation.

Biofeedback and direct intervention through muscle stimulation has also been used alongside traditional physical therapy manual interventions with an aim to reduce pain and improve outcomes. Electromyography can be used as a way of providing biofeedback during exercise, but showed conflicting results in the studies identified with some suggesting benefits and others no difference. NMES has been used to replicate the effects of physical therapy strengthening without the need to perform dynamic loadbearing movements, which are often painful for KOA patients, however this research showed conflicting results in terms of effectiveness. NMES research is a mature area and our focus on recently applied technology in KOA (with the 10-year window of 2010–2020) does not fully capture the substantial literature base around the generic use of NMES, but highlights a comparative paucity of recent application, suggesting perhaps a waning enthusiasm for this particular modality in KOA.

Several biofeedback systems have been developed for the purpose of altering gait to reduce pain or slow disease progression. As such, these systems provide biofeedback to encourage patients to alter their movement patterns. The technologies identified for this application include force transducers, sensory feedback systems, camera systems, inertial measurement units and virtual reality systems. Most of the research papers we identified assessed whether these systems could reduce knee joint loads. Several systems showed that loads could be reduced through biofeedback, however, these technologies are currently restricted to use within a laboratory environment, which does not translate to a real-world intervention for KOA patients, and the long-term maintenance of the altered gait patterns post-intervention not well established. Force transducers, IMUs and virtual reality systems can though provide a method of altering gait patterns in everyday settings, through use in shoes or in walking aids. Thus far, these technologies have been employed in isolation and few studies have described a ‘complete’ system replicating the gait-lab that provides feedback on movement patterns based on an objective measure of force. The use of relatively simple remote sensor systems did show the capability to provide feedback, which resulted in reduced loads, however the long-term impact of adopting these altered gait patterns is unknown and the systems need further refinement to enable seamless integration within the individuals with KOA’ daily life.

We found clear examples of technology used within current lifestyle interventions with patients but primarily as measurement tools or a way of managing physical therapy consultations (tele-rehabilitation). There is a surprising lack of clinical data as to whether combining sensory feedback to a patient in everyday life actually makes an impact on their clinical or physical outcomes. Why is it so hard to get patient level data fed back to clinicians to be able to alter programmes or change delivery? Simply using existing technologies offers opportunities to develop lifestyle interventions that combine evidence-based telepresence research with new and emerging biofeedback or IMU driven objective measures. This could transform the delivery of ‘remote’ therapy consultations, moving away from a video call to an active therapy session where real-time data is collected, processed, and interpreted to facilitate the intervention. There remains however a substantial challenge to the engineering and clinical community to develop systems that offer the clinician feedback so as to be able to alter the individual's treatment pathway.

Perhaps unsurprisingly, the studies looking at development in healthy populations tended to explore more advanced technology or the integration of multiple systems compared to the work reported in clinical rehabilitation applications with patients. There does seem to be something of a shift in development towards more advanced technologies such as conductive textiles, along with the increased investigation of virtual reality feedback and algorithms for interpreting data. Despite the welcome exploration of these advanced tools within KOA, the field remains firmly at a developmental stage, with seemingly little translation into clinical arenas. It also appears that the purpose of technological development is for the same basic applications as are currently employed. For example, the papers investigating conductive textiles were exploring how the technology could assess physical therapy exercises or measure foot pressure using the piezoresistive properties of the textiles. Ultimately this tool would be used within a multi-model tele-rehabilitation system or gait rehabilitation system.

We also note the seeming lack of co-creation of technologies and/or technology enabled interventions with KOA patients. A single paper in the 78 we include in this review described the piloting of a system with physical therapists, however patient involvement in technology development is conspicuously lacking. Clearly the patient must be at the forefront of developing such technology to ensure the tools and interventions are fit for purpose.

### 4.1 Strengths and limitations

Strengths of this scoping review are the substantial search conducted across eight databases and resultant overview of the use of technology within KOA lifestyle interventions. Limitations include the restricted timeframe data ranges, purposely chosen to reflect recent developments, but that may have missed reports of some more established interventions, and that the information provided is a top-level overview and synthesis as opposed to a methodological critique of the included papers, this though is the accepted output of scoping reviews. We focussed this review on scoping the technology available for direct use with clinical cohorts and technology enabled interventions. As such we have not considered here the uptake of this technology amongst clinical cohorts. This reflects the early stage as to the technology transfer we describe.

### 4.2 Conclusions

The predominant current use of technology in OA lifestyle interventions is through well-established telecommunication and commercially available measurement devices, while integrating new advanced technologies seems a longer-term goal. This scoping review perhaps demonstrates that the translation of technology from healthy participant research to clinical interventions is slow.

## Contributions

All authors made substantial contribution to the article. The study was conceived by DFH and RKJ and designed by DFH, YP and RKJ. SA and BS collected and collated the data. All authors were involved in interpreting the data. DFH, drafted the final manuscript with contribution from all authors. All approved the final version.

## Funding

This work was funded by the 10.13039/501100000266EPSRC/OATech Network Plus [EP/N027264/1]. The funders played no role in the design, collection, analysis, and interpretation of results or writing of manuscript.

## Declaration of competing interest

The authors declare that they have no known competing financial interests or personal relationships that could have appeared to influence the work reported in this paper.
